# Complete Genome Sequence of Providencia stuartii CMC-4104, Isolated from a Human Splenic Abscess, Containing Multiple Copies of NDM-1 and PER-1 Carbapenem Resistance Genes

**DOI:** 10.1128/mra.00514-22

**Published:** 2022-08-04

**Authors:** Jayasimha Rao, Nicholas K. Stornelli, Nathan A. Everson, Lauren F. McDaniel, Mariana Gomez De La Espriella, Jason R. Faulhaber, S. Michelle Todd, Kevin K. Lahmers, Roderick V. Jensen

**Affiliations:** a Internal Medicine, Division of Infectious Disease, Carilion Medical Center, Roanoke, Virginia, USA; b Division of Infectious Disease, Virginia Tech Carilion School of Medicine, Roanoke, Virginia, USA; c Center for Emerging, Zoonotic, and Arthropod-Borne Pathogens, Virginia Tech, Blacksburg, Virginia, USA; d Department of Pharmacy, Carilion Roanoke Memorial Hospital, Roanoke, Virginia, USA; e Department of Biomedical Sciences and Pathobiology, VA-MD College of Veterinary Medicine, Virginia Tech, Blacksburg, Virginia, USA; f Department of Biological Sciences, Virginia Tech, Blacksburg, Virginia, USA; University of Maryland School of Medicine

## Abstract

We report the complete genome sequence of a clinical isolate of Providencia stuartii strain CMC-4104, isolated from a splenic abscess. Oxford Nanopore Technologies (ONT) and Illumina sequencing reads were assembled using Geneious to generate a 4,504,925-bp circular chromosome containing multiple copies of the NDM-1 and PER-1 genes in a genomic resistance island.

## ANNOUNCEMENT

The spread of carbapenemase-producing *Enterobacterales* is increasing in health care facilities (1). We describe a multidrug-resistant (MDR) clinical isolate of Providencia stuartii recovered from an infected necrotizing pancreatitis patient, belonging to the class of difficult-to-treat resistance (2, 3) strains, panresistant to first-line antimicrobials, including newer β-lactam/β-lactamase inhibitor combinations (4–6).

P. stuartii CMC-4104, isolated from a patient with splenic abscess aspirate, was inoculated onto sheep blood agar and MacConkey agar plates and incubated at 35°C aerobically for 24 to 72 h. Antibiotic susceptibility testing for 17 antimicrobial agents based on the CLSI M100 standards (7) was carried out by Quest Diagnostics at the Carilion Clinic (Table 1).

**TABLE 1:** Antimicrobial susceptibility for P. stuartii strain CMC-4104

Antimicrobial	MIC^*a*^	Result
Trimethoprim sulfamethoxazole	≥320	R
Ampicillin+sulbactam	≥32	R
Ertapenem	≥8	R
Imipenem	≥16	R
Piperacillin+tazobactam	≥128	R
Cefazolin	≥64	R
Cefepime	≥32	R
Ceftazidime	≥64	R
Ceftriaxone	≥64	R
Ceftazidime+avibactam^*b*^	≥250	R
Imipenem+relebactam^*b*^	≥32	R
Meropenem+vaborbactam^*b*^	≥250	R
Tobramycin/gentamicin	4	R
Levofloxacin	≥16	R
Cefiderocol^*c*^	2	S

a MICs were determined (7) using the Vitek and Verigene testing systems at the Quest Diagnostics microbiology laboratory at Carilion. The carbapenemase-producing strain was confirmed by the Commonwealth of Virginia Consolidated Laboratory Services, VDH.

b Susceptibility testing was performed using the E-test for ceftazidime+avibactam, imipenem+relebactam, and meropenem+vaborbactam.

c Cefiderocol susceptibility testing was completed by Associated Regional and University Pathologists Laboratories in Salt Lake City, UT.

A single colony of P. stuartii CMC-4104 was grown in 25 mL lysogeny broth at 37°C and 200 rpm for 18 h. The cell pellet was used for genomic DNA isolation with a Genomic-tip 20/G kit (8, 9). The unsheared DNA was sequenced without size selection using the ONT 9.4.1 MinION flow cell with the SQK-LSK109 ligation sequencing kit. MiSeq Illumina paired-end sequencing (350-bp insert size) was performed using the TruSeq DNA PCR-free library prep kit.

The ONT sequencing generated 19,109 reads with a maximum length of 170,418 bp and an average length of 15,700 bp for a total of 306 Mbp. Base calling was performed using Guppy 4.4.1, and Porechop 0.2.4 (10) was used for adaptor trimming. The resulting fastq files were initially assembled using Flye 2.8 (11) in Geneious Prime 2022.0.2 to produce a large, closed 4.59-Mbp draft genome with ~65× coverage. The Illumina sequencing generated 2,290,766 paired-end reads 150 bp long for a total of 344 Mbp. The “Map to Reference” tool in Geneious Prime was used (with low sensitivity settings) to align all of the Illumina reads to the draft genome sequence and correct ONT sequencing errors. Variations in the Illumina read coverage were used to identify three circular plasmid sequences and a highly repeated set of MDR gene cassettes in the main chromosome. Finally, Geneious Prime was used to align the ONT reads to the plasmid and genome sequences (with medium sensitivity settings) to confirm closure of the circular plasmid and main chromosome sequences, as well as the repetition of the MDR gene cassettes. Default parameters were used for all software unless otherwise specified.

**FIG 1:**
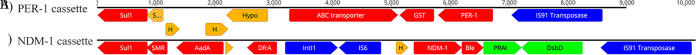
Gene organization in the repeated multidrug-resistant (MDR) cassettes. (A) The PER-1 cassette (8,986 bp) included genes for sulfonamide-resistant dihydropteroate synthase (*sul1*), small multidrug resistance (SMR) (*qacE*Δ1) (truncated), hypothetical proteins, ATP-binding protein/permease (ABC transporter), glutathione *S*-transferase (GST) family protein, class A extended-spectrum β-lactamase (*bla*_PER-1_), and IS*CR1* family transposase (IS*91*). (B) The New Delhi metallo β-lactamase (*bla*_NDM-1_) cassette (10,494 bp) included genes for sulfonamide-resistant dihydropteroate synthase (*sul1*), small multidrug resistance (SMR) *qacE*Δ1, aminoglycoside 3″-*O*-nucleotidyltransferase (*aadA*), DUF1010 domain-containing hypothetical protein, trimethoprim-resistant dihydrofolate reductase (*dfrA*12), class 1 integron integrase (*intI*1), IS*26* family transposase (IS*6*), IS*30* family transposase (truncated), subclass B1 β-lactamase (*bla*_NDM-1_), bleomycin resistance protein (Ble), phosphoribosylanthranilate isomerase (PRAI), cytochrome c-type biogenesis protein/protein-disulfide reductase (*dsbD*), and IS*CR1* family transposase (IS*91*). Red represents drug resistance genes; blue, transposases; green, metabolic genes; and yellow, hypothetical or pseudogenes (truncated or overlapped). Annotated genes were used from NCBI GenBank, and the scale indicates the number of base pair residues.

Final assembly of the P. stuartii CMC-4104 genome resulted in a main chromosome (GenBank accession number CP095443) of 4,504,925 bp with 41.4% GC content and an average Illumina coverage (AIC) of 65×; a large, low-copy, circular plasmid (CP095444) of 278,489 bp with 47.3% GC content and an AIC of 85×; a small, high-copy, circular plasmid (CP095445) of 2,683 bp with 41.8% GC content and an AIC of 4,616×; and a phage-like circular sequence (CP095442) of 51,458 bp with 41.7% GC content and an AIC of 105×. In the main chromosome (CP095443), the NCBI Prokaryotic Genome Annotation Pipeline (PGAP) 6.1 (12) was used to identify 4,066 protein coding genes, 79 tRNAs, 22 rRNAs, and 4 CRISPR arrays. Of particular note was a large 215-kbp genomic resistance island (*Ps*GRI) integrated with a site-specific integrase into the 3′ end of a tRNA modification GTPase gene (*mnmE*), like the Salmonella genomic island (13, 14). Remarkably, the *Ps*GRI contained 15 copies of the MDR gene cassettes containing *bla*_NDM-1_ and 2 copies of *bla*_PER-1_ shown in Fig. 1. The large plasmid (CP095444) also contained one complete copy each of the *bla*_NDM-1_ and *bla*_PER-1_ MDR cassettes and an additional *bla*_OXA-10_ MDR cassette (14–18). The small, high-copy plasmid (CP095445) also contained a fluoroquinolone resistance gene, *qnrD* (19). The assembly revealed a circular phage-like sequence (CP095442) that is identical to a prophage in the main chromosome and is very similar to a second prophage integrated into a CRISPR locus (20).

### Data availability.

The annotated complete genome assembly of strain Providencia stuartii CMC-4104 is available at GenBank under accession numbers CP095442.1, CP095443.1, CP095444.1, and CP095445.1, SRA accession numbers SRR18691816 and SRR18691817, BioProject accession number PRJNA824933, and BioSample accession number SAMN27484493.

## References

[1] Murray CJ, Ikuta KS, Sharara F, Swetschinski L, Robles Aguilar G, Gray A, Han C, Bisignano C, Rao P, Wool E, Johnson SC, Browne AJ, Chipeta MG, Fell F, Hackett S, Haines-Woodhouse G, Kashef Hamadani BH, Kumaran EAP, McManigal B, Agarwal R, Akech S, Albertson S, Amuasi J, Andrews J, Aravkin A, Ashley E, Bailey F, Baker S, Basnyat B, Bekker A, Bender R, Bethou A, Bielicki J, Boonkasidecha S, Bukosia J, Carvalheiro C, Castañeda-Orjuela C, Chansamouth V, Chaurasia S, Chiurchiù S, Chowdhury F, Cook AJ, Cooper B, Cressey TR, Criollo-Mora E, Cunningham M, Darboe S, Day NPJ, De Luca M, Dokova K, et al. 2022. Global burden of bacterial antimicrobial resistance in 2019: a systematic analysis. Lancet 399:629–655. doi:10.1016/S0140-6736(21)02724-0. 35065702 PMC8841637

[2] Abdallah M, Balshi A. 2018. First literature review of carbapenem-resistant *Providencia*. New Microbes New Infect 25:16–23. doi:10.1016/j.nmni.2018.05.009. 29983987 PMC6031241

[3] Kadri SS, Adjemian J, Lai YL, Spaulding AB, Ricotta E, Prevots DR, Palmore TN, Rhee C, Klompas M, Dekker JP, Powers JH, Suffredini AF, Hooper DC, Fridkin S, Danner RL, National Institutes of Health Antimicrobial Resistance Outcomes Research Initiative (NIH–ARORI). 2018. Difficult-to-treat resistance in Gram-negative bacteremia at 173 US hospitals: retrospective cohort analysis of prevalence, predictors, and outcome of resistance to all first-line agents. Clin Infect Dis 67:1803–1814. doi:10.1093/cid/ciy378. 30052813 PMC6260171

[4] Coppi M, Di Pilato V, Monaco F, Giani T, Conaldi PG, Rossolini GM. 2020. Ceftazidime-avibactam resistance associated with increased bla KPC-3 gene copy number mediated by pKpQIL plasmid derivatives in sequence type 258 *Klebsiella pneumoniae*. Antimicrob Agents Chemother 64:e01816-19. doi:10.1128/AAC.01816-19. 31964792 PMC7179273

[5] Wang Y, Wang J, Wang R, Cai Y. 2020. Resistance to ceftazidime-avibactam and underlying mechanisms. J Glob Antimicrob Resist 22:18–27. doi:10.1016/j.jgar.2019.12.009. 31863899

[6] Lebreton F, Corey BW, McElheny CL, Iovleva A, Preston L, Margulieux KR, Cybulski RJ, Mc Gann P, Doi Y, Bennett JW. 2021. Characterization of KPC-82, a KPC-2 variant conferring resistance to ceftazidime-avibactam in a carbapenem-nonsusceptible clinical isolate of *Citrobacter koseri*. Antimicrob Agents Chemother 65:e0015021. doi:10.1128/AAC.00150-21. 33972237 PMC8218621

[7] Clinical and Laboratory Standards Institute. 2020. Performance standards for antimicrobial susceptibility testing, 30th ed. Document M100. CLSI, Wayne, PA.

[8] Adenikinju A, Jensen RV, Kerkering TM, Garner DC, Rao J. 2020. Complete genome sequence of *Pseudompnas aeruginosa* CMC-115, a clinical strain from an acute ventilator-associated pneumonia patient. Microbiol Resour Announc 9:e00595-20. doi:10.1128/MRA.00595-20. 32703835 PMC7378034

[9] Rao J, Susanti D, Childress JC, Mitkos MC, Brima JK, Baffoe-Bonnie AW, Pearce SN, Grgurich D, Fernandez-Cotarelo MJ, Kerkering TM, Mukhopadhyay B. 2018. Tn2008-driven carbapenem resistance in *Acinetobacter baumannii* isolates from a period of increased incidence of infections in a southwest Virginia hospital (USA). J Glob Antimicrob Resist 12:79–87. doi:10.1016/j.jgar.2017.08.017. 28899807

[10] Wick RR, Judd LM, Gorrie CL, Holt KE. 2017. Completing bacterial genome assemblies with multiplex MinION sequencing. Microb Genom 3:e000132. doi:10.1099/mgen.0.000132. 29177090 PMC5695209

[11] Kolmogorov M, Yuan J, Lin Y, Pevzner PA. 2019. Assembly of long, error-prone reads using repeat graphs. Nat Biotechnol 37:540–546. doi:10.1038/s41587-019-0072-8. 30936562

[12] Li W, O'Neill KR, Haft DH, DiCuccio M, Chetvernin V, Badretdin A, Coulouris G, Chitsaz F, Derbyshire MK, Durkin AS, Gonzales NR, Gwadz M, Lanczycki CJ, Song JS, Thanki N, Wang J, Yamashita RA, Yang M, Zheng C, Marchler-Bauer A, Thibaud-Nissen F. 2021. RefSeq: expanding the Prokaryotic Genome Annotation Pipeline reach with protein family model curation. Nucleic Acids Res 49:D1020–D1028. doi:10.1093/nar/gkaa1105. 33270901 PMC7779008

[13] de Curraize C, Siebor E, Neuwirth C. 2021. Genomic islands related to *Salmonella* genomic island 1; integrative mobilisable elements in trmE mobilised in trans by A/C plasmids. Plasmid 114:102565. doi:10.1016/j.plasmid.2021.102565. 33582118

[14] Siebor E, de Curraize C, Neuwirth C. 2019. Identification of AGI1-A, a variant of Acinetobacter genomic island 1 (AGI1), in a French clinical isolate belonging to the *Enterobacter cloacae* complex. J Antimicrob Chemother 74:311–314. doi:10.1093/jac/dky442. 30412264

[15] Carraro N, Rivard N, Burrus V, Ceccarelli D. 2017. Mobilizable genomic islands, different strategies for the dissemination of multidrug resistance and other adaptive traits. Mob Genet Elements 7:1–6. doi:10.1080/2159256X.2017.1304193. PMC5397120 28439449

[16] McGann P, Hang J, Clifford RJ, Yang Y, Kwak YI, Kuschner RA, Lesho EP, Waterman PE. 2012. Complete sequence of a novel 178-kilobase plasmid carrying bla(NDM-1) in a Providencia stuartii strain isolated in Afghanistan. Antimicrob Agents Chemother 56:1673–1679. doi:10.1128/AAC.05604-11. 22290972 PMC3318346

[17] Shin S, Jeong SH, Lee H, Hong JS, Park MJ, Song W. 2018. Emergence of multidrug-resistant *Providencia rettgeri* isolates co-producing NDM-1 carbapenemase and PER-1 extended-spectrum β-lactamase causing a first outbreak in Korea. Ann Clin Microbiol Antimicrob 17:20. doi:10.1186/s12941-018-0272-y. 29728111 PMC5935979

[18] Shen S, Huang X, Shi Q, Guo Y, Yang Y, Yin D, Zhou X, Ding L, Han R, Yu H, Hu F. 2021. Occurrence of NDM-1, VIM-1, and OXA-10 co-producing *Providencia rettgeri* clinical isolate in China. Front Cell Infect Microbiol 11:789646. doi:10.3389/fcimb.2021.789646. 35047418 PMC8761753

[19] Guillard T, Grillon A, de Champs C, Cartier C, Madoux J, Berçot B, Lebreil A-L, Lozniewski A, Riahi J, Vernet-Garnier V, Cambau E. 2014. Mobile insertion cassette elements found in small non-transmissible plasmids in Proteeae may explain qnrD mobilization. PLoS One 9:e87801. doi:10.1371/journal.pone.0087801. 24504382 PMC3913671

[20] Varble A, Campisi E, Euler CW, Maguin P, Kozlova A, Fyodorova J, Rostøl JT, Fischetti VA, Marraffini LA. 2021. Prophage integration into CRISPR loci enables evasion of antiviral immunity in *Streptococcus pyogenes*. Nat Microbiol 6:1516–1525. doi:10.1038/s41564-021-00996-8. 34819640

